# The Effects of Acute Sleep Curtailment on Salt Taste Measures and Relationships with Energy-Corrected Sodium Intake: A Randomized Cross-Over Trial with Methodology Validation

**DOI:** 10.3390/ijerph20054140

**Published:** 2023-02-25

**Authors:** Chen Du, Russell Keast, Sze-Yen Tan, Robin M. Tucker

**Affiliations:** 1 Department of Food Science and Human Nutrition, Michigan State University, East Lansing, MI 48824, USA; 2 CASS Food Research Centre, School of Exercise and Nutrition Sciences, Deakin University, Geelong, VIC 3220, Australia; 3 Institute for Physical Activity and Nutrition (IPAN), School of Exercise and Nutrition Sciences, Deakin University, Geelong, VIC 3220, Australia

**Keywords:** salt taste, intensity, preference, salt-liking phenotype, diet, sleep

## Abstract

(1) Background: Sleep may be a factor that influences the taste–dietary intake relationship. The effect of sleep on salt taste measures has not been adequately studied, and no standardized methodology has been developed for measuring salt taste preference. (2) Methods: A sweet taste forced-choice paired-comparison test was adapted and validated to determine salt taste preference. In a randomized cross-over trial, participants slept a curtailed night (33% reduction in sleep duration) and a habitual night, confirmed by a single-channel electroencephalograph. Salt taste tests were conducted the day after each sleep condition using five aqueous NaCl solutions. One 24-h dietary recall was obtained after each taste test. (3) Results: The adapted forced-choice paired-comparison tracking test reliably determined salt taste preference. No changes in salt taste function (intensity slopes: *p* = 0.844) or hedonic measures (liking slopes: *p* = 0.074; preferred NaCl concentrations: *p* = 0.092) were observed after the curtailed sleep condition compared to habitual sleep. However, sleep curtailment disrupted the association between liking slope and energy-corrected Na intake (*p* < 0.001). (4) Conclusions: The present study serves as the first step toward more standardized taste assessments to facilitate comparison between studies and suggests accounting for sleep when exploring taste–diet relationships.

## 1. Introduction

Although taste plays a critical role in determining dietary intake [1,2,3], laboratory-based taste measurements are often unreliable predictors of intake [4,5]. Using salt taste as an example, some studies reported that the hedonic measures, including liking and preference, of salty stimuli were positively correlated with dietary salt and salty food intake [6,7,8], while other studies reported that salt liking did not predict dietary salt intake [9,10]. Relationships between diet and measures of salt taste function, such as taste thresholds and intensity ratings, are also varied; some studies report that these measures do not predict intake [11,12,13,14,15,16], while others report that they do [17,18,19,20]. Given the conflicting findings, exploring factors that may influence the taste–diet relationship is warranted.

Sleep is one physiological process that has been shown to alter measures of sweet taste [21,22] as well as dietary intake [23,24,25]. Increases in preferred concentrations for sucrose [21,26] and sucralose were observed after one night of curtailed sleep [21]. When testing sweet taste using solid foods, sweeter oat crisps were liked more after curtailed sleep compared to habitual sleep [26]. In summary, one night of short sleep altered hedonic measures of sweet taste, which can lead to undesirable dietary behaviors such as increased consumption of sweets and high calorie foods [24,27,28,29,30].

While alterations in sweet taste after sleep curtailment have been demonstrated, there is limited investigation into the effects of sleep on salt taste. One cross-sectional study reported an association between short sleep duration (defined as <6 h) and increased odds of self-reported altered taste perception in general [31]. A separate study demonstrated self-reported sleepiness was not associated with salt taste sensitivity, but positively associated with cravings for savory foods [32]. Two experimental studies investigated the effects of complete absence of sleep (sleep deprivation) and shortened sleep duration (sleep curtailment) on salt taste function. One study reported no change in salt taste detection threshold after 24, 48, and 72 h of sleep deprivation [33], while the other study demonstrated no differences in salt taste sensitivity between individuals with long (>7 h) versus short (<7 h) sleep [34]. To our knowledge, these are the only investigations that investigated the effects of sleep on salt taste measures.

Apart from limited studies in this area, there are also several limitations in these previous studies that investigated the effects of sleep on salt taste measures. First, none of the studies included hedonic measures of salt taste, which are more reliable predictors of dietary intake than thresholds [4,5]. Second, the complete absence of sleep is not commonly experienced in the general adult population [35,36]. Third, the study that compared short versus long sleep duration did not prescribe the curtailed sleep duration based on the participants’ habitual sleep duration, which could have created an unequal sleep curtailment duration for individuals, i.e., some individuals were curtailed more or less than others. This methodological limitation could reduce the ability to detect differences, should they exist. Given the small number of studies and these limitations, research investigating the effects of sleep curtailment on both hedonic and functional measures of salt taste is needed.

To address the lack of understanding regarding salt taste hedonics, it is important to develop a method that enables the classification of adults based on their salt-liking phenotypes, e.g., likers and dislikers. This is important, as previous work suggests that sweet-liking phenotype and sleep curtailment interact [21,22,26,37]. Sweet-liking phenotypes are used to broadly categorize individuals as sweet likers or dislikers, and have been repeatedly identified in the literature by our group [22,26,37,38] and others, e.g., [39,40,41]. Whether salt phenotypes are identifiable and whether they influence taste responses after sleep curtailment have not been previously explored.

The present work includes two components: part one validated a salt taste preference evaluation method, and part two applied this method in an experiment that investigates the effect of salt taste curtailment on salt taste preference. Four aims were addressed: First, since a standardized salt preference test does not exist [4], the methodology validation component of the study aimed to examine whether an adapted version of a forced-choice paired-comparison test developed for sweet taste [42] could be used in determining salt taste preference (aim 1). Part two investigated the effects of sleep curtailment on salt taste hedonic measures (liking and preferred concentration) and function (intensity) (aim 2); examined whether any of the taste tests were associated with energy-corrected sodium intake (aim 3); and explored whether identifiable salt-liking phenotypes were present (aim 4). The researchers hypothesized that:

1. The adapted forced-choice paired-comparison tracking test could serve as a valid and reliable tool to determine salt taste preference.
2. Salt taste liking, measured by the slope of liking ratings of five salty solutions across different concentrations would be steeper after a night of curtailed sleep compared to a night of habitual sleep; however, salt taste function, measured by the slope of intensity ratings would not be affected. Additionally, preferred salt concentration, measured by the newly developed test, would increase.
3. Hedonic measures of salt taste (liking slope and preferred salt concentration) and salt taste function (intensity slope) would be positively associated with energy-corrected Na intake after both the habitual and the curtailed night of sleep.
4. Distinguishable salt-liking phenotypes would be identified.

## 2. Materials and Methods

### 2.1. Participants

Participants, including both males and females (*n* = 59), between the ages of 18 and 45 years with no diagnosed sleep conditions who reported habitually sleeping 7 to 9 h per night and had regular weekday bedtimes were recruited for the study. Potential participants who had conditions that may affect taste function and dietary intake, such as type 2 diabetes and cardiovascular diseases, were excluded from the study. A screening questionnaire was used to check eligibility. Sleep quality, measured with the Pittsburg Sleep Quality Index (PSQI), was examined at screening for recruiting approximately equal numbers of good and poor sleepers [43]. PSQI scores range from 0 to 21 with higher scores indicating worse sleep quality; scores ≥ 5 indicate poor sleep quality, while scores < 5 indicate good sleep quality.

### 2.2. Study Protocol and Timeline

This was a randomized, crossover study that included a consent visit, plus two lab visits after a night of habitual and curtailed sleep in random order. The two lab visits were at least seven days apart to provide a washout period to recover from the one night of shortened sleep. After each lab visit, participants were asked to fill out one 24-h dietary recall the following morning using the Automated Self-Administered 24-h (ASA24^®^) Dietary Assessment [44] to record everything consumed during the day of the taste test, including meals, snacks, and beverages. ASA24^®^ has been validated and widely used in clinical studies [44,45,46,47].

### 2.3. Compliance to Sleep Protocol

For the sleep protocol, participants were instructed to wear the Zmachine (General Sleep, Columbus, OH, USA), a single channel electroencephalogram (EEG) that monitors sleep duration and stages, during sleep on both nights. The Zmachine has been validated against polysomnography (PSG) [48] and is widely used in sleep research studies [22,26,30,49]. Participants were instructed to put the Zmachine on 30 min before going to bed. For the habitual night, bedtime and waketime were determined based on typical self-reported bed and wake times while curtailed bed and wake times were calculated by reducing self-reported total habitual sleep time by 33% and delaying bedtime [22,26]. For example, one participant reported typically going to bed at 10:00 p.m. and waking up at 7:00 a.m. The total bedtime for the participant was 9 h. For the curtailed night of sleep, a 3-h reduction of sleep (33% of reduction in sleep duration) was applied, and the bed and wake times were 1:00 a.m. and 7:00 a.m., respectively. If a participant did not follow the sleep protocol they were assigned, they were asked to repeat the protocol.

### 2.4. Consent Visit

During the consent visit, eligible participants completed the demographic questionnaire and were measured for weight, height, and percent body fat (%BF). Demographic questions included gender, race, ethnicity, and age. The height of participants was measured using a standing stadiometer (HM200P, Charder, Taichung, Taiwan). Weight and %BF were evaluated using a bioelectrical impedance scale (TBF-400, Tanita, Arlington Heights, IL, USA).

### 2.5. Lab Visits

Participants returned to the lab after one night of curtailed sleep and one night of habitual sleep. These two lab visits were identical. At each visit, the sleep recording data were reviewed in the Zmachine data viewer for each participant prior to taste testing to confirm that the participant followed the intended (habitual vs. curtailed) sleep protocol. Participants who did not follow the sleep protocol, which was defined as more than a 30-min discrepancy between actual sleep time and protocol determined sleep time, were asked to repeat a night of sleep following the relevant protocol at least a week later. After confirmation that the participant had adhered to the assigned protocol for that night, they were asked how they would rate the previous night’s sleep quality and duration on a visual analog scale (VAS) of 0 to 100. Zero indicated far below average while 100 indicated far above average.

### 2.6. Taste Testing

The five NaCl solutions presented to participants included concentrations of 0.05, 0.09, 0.15, 0.19, and 0.25 M NaCl, which were selected to reflect the spectrum of salt taste in a real-world food environment [50]. For example, the lowest concentration salt solution (0.05 M) represents the salt concentration of milk, while the most concentrated salt solution (0.25 M) reflects the salt concentration of pickle juice. Concentrations were pilot tested in the laboratory prior to the study to reflect commonly experienced dietary salt exposures [50] and to ensure they were distinguishable from each other.

First, five 10 mL salty solutions made with NaCl and distilled water in different concentrations were presented to participants in 30 mL plastic portion cups, in order of increasing concentration (0.05, 0.09, 0.15, 0.19, and 0.25 M). Participants were asked to put each solution in their mouth and swish for as long as needed to thoroughly evaluate the solution and then expectorate. Next, participants were asked to rate the liking of each solution on a VAS of 0 to 100, where 0 reflected ‘not at all’ and 100 represented ‘extremely.’ Immediately after the liking task, participants were asked how intense they thought the salty solutions were on a VAS of 0 to 100, where 0 indicated ‘not at all’ and 100 signified ‘extremely.’ After rating their liking and intensity, participants were asked to rinse their mouth with distilled water after each sample until no saltiness was perceived. After 30 s, the next sample was tasted.

The preferred salt concentration test followed examination of liking and intensity. This test adapted the two-series forced-choice paired-comparison tracking procedure developed by Mennella et al. (2011) [42]. The forced-choice paired-comparison tracking procedure has been validated in determining preferred sweet concentrations [42]. The same 5 concentrations of aqueous NaCl solutions as those used for the liking and intensity tests were used. In the first series of tests, participants were presented a pair of salt solutions, lower concentration first, and asked to taste both solutions while rinsing in between with distilled water. After tasting solutions in pairs, participants identified their preferred salt solution. As with the sweet taste forced-choice paired-comparison tracking protocol, the 0.09 M and 0.19 M NaCl solutions, were presented to participants first for each trial. Then, each subsequent pair presented contained the participants’ previously preferred concentration paired with an adjacent solution, either higher or lower in concentration. At each presentation, the lower concentration was presented first. The tests were repeated until one preferred concentration was sequentially selected twice while the adjacent concentrations to the preferred concentration had been tasted. This preferred concentration was recorded as the preferred concentration for trial 1. Participants were given two minutes to rest in between trial 1 and the second trial to avoid fatigue. For trial 2, the series of tests were repeated; however, the higher concentration of each pair of solutions were presented to participants first to reduce the possibility of an order effect [42]. The preferred concentration for the second series of tests was recorded as the preferred concentration for trial 2. The geometric mean of the preferred concentration for trial 1 and 2 was calculated for each participant to avoid position bias and improve the accuracy in estimating the preferred concentration [42].

### 2.7. Statistical Analysis

Descriptive statistics were performed. Variables are presented as mean ± standard deviations unless specified otherwise. Sample size calculations were performed based on a power of 0.8, an alpha of 0.05, and a median effect size of 0.5. A total sample size of 40 participants was required to achieve 80% power. The geometric mean of preferred salt concentrations from trial 1 and 2 under each sleep condition was calculated and used in analyses. Paired t-tests were used to determine the differences of salt taste function (slope of intensity ratings), preference (slope of liking ratings and preferred concentration), sodium intake, energy-corrected sodium intake, macronutrient intake between the curtailed and the habitual night, after confirming the linearity of these variables were met. Additionally, time in bed, total sleep time, deep sleep, and REM sleep were compared between the curtailed and the habitual night of sleep to verify that the sleep protocol was implemented correctly. To examine whether the adapted forced-choice paired-comparison tracking test is a valid tool for determining salt taste preference, only data from the habitual night of sleep was used. First, the geometric means of preferred salt concentration were compared between visit 1 and visit 2 using one-way ANOVA to ensure no order effect was present. Then, the intensity rating of each concentration of NaCl solution was compared to all others using paired t-tests [42], and a false discovery rate (FDR) of q = 0.05 was employed to reduce the risk of Type 1 error. The geometric mean of preferred salt concentrations for trial 1, trial 2, and the overall sample were compared between each other. In addition, bivariate correlations were used to examine the associations between the liking slope and the preferred salt concentration. Additionally, hierarchical cluster analysis with between groups linkage was performed to identify salt-liking phenotypes using the habitual sleep data only, as this night reflects typical behavior. Further, a zero-order Pearson correlation matrix was created to examine the relationships between age, BMI, %BF, PSQI, total sleep time, liking slope, intensity slope, preferred salt concentration, and energy-corrected Na intake. FDR was again used to correct for multiple comparisons. To explore whether variabilities in salt-liking ratings influenced the relationship between liking and intake under different sleep conditions, the variance of liking ratings for each solution was compared between the habitual and the curtailed condition using the Levene test, which is commonly used to determine differences in variances between samples [51,52]. Data analysis was completed using SPSS version 27 (IBM Corporation, Armonk, NY, USA). *p* < 0.05 was used to determine statistical significance in all analyses.

## 3. Results

### 3.1. Anthropometric and Demographic Information

A total of 59 participants complied with the sleep protocols and completed the study. More than two-thirds of the participants were female, nearly half were white, more than one-third were Asian, and the average BMI was considered to be in the healthy range (Table 1).

### 3.2. Validation of the Adapted Forced-Choice Paired-Comparison Tracking Test in Determining Salt Taste Preference

Analyses indicated that the preferred salt concentration was not different between those who experienced the habitual night first and those who experienced the habitual night second. This result confirmed that no order effect was detected (Table 2).

**Figure 1:**
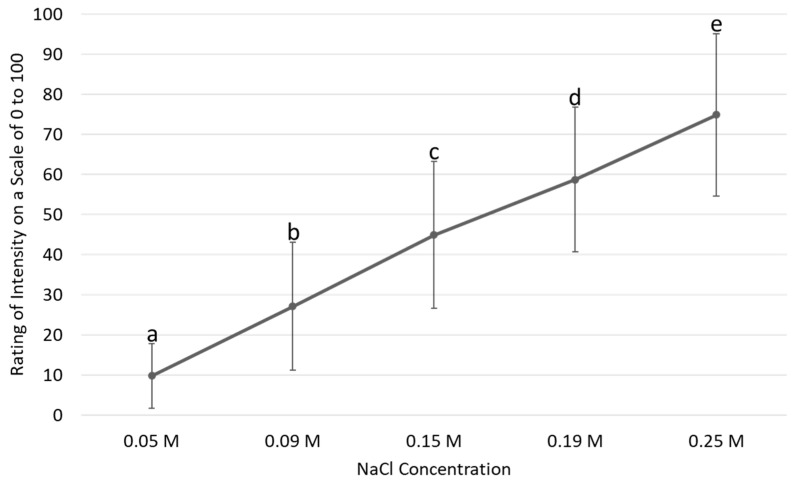
Mean intensity ratings of salt solutions across all concentrations on the habitual sleep night. Note: Different letters indicate significant differences between concentrations.

To evaluate the utility and reliability of the adapted procedure, it was necessary to first confirm that all salt stimuli concentrations were perceptibly different in terms of intensity. This was confirmed (*p* < 0.001 for all concentration comparisons, Figure 1).

Next, preferred concentrations across the two trials were compared and no significant difference was found between the two sleep conditions; that is, the preferred salt concentration determined in Trial 1 was not different from Trial 2 (*p* = 0.078) or from the geometric mean of the two trials (*p* = 0.948). The concentration of Trial 2 also did not differ from the geometric mean (*p* = 0.089). Out of the 59 participants, 48 (82%) participants either selected the same concentration for Trial 1 and 2 (*n* = 27, 46%) or selected neighboring concentrations in Trial 1 and 2 (*n* = 21, 36%). To further demonstrate the validity of this method, the preferred salt concentration was positively and moderately to strongly correlated with the liking slope of the five test solutions (r = 0.593, *p* < 0.001).

### 3.3. Compliance of the Sleep Protocol

As intended, the time in bed as well as total sleep, slow wave sleep (SWS), and REM sleep times were different between the habitual and the curtailed nights (Table 3). Additionally, the reduction of total sleep time from the habitual to the curtailed night was 36.1%, which confirmed that the 33% sleep duration reduction was achieved, indicating that the sleep protocol for each night was implemented correctly by participants.

### 3.4. No Difference in Salt Taste Hedonic Measures and Function, Sodium and Macronutrient Intake, and Food Cravings between the Curtailed and Habitual Nights

**Figure 2:**
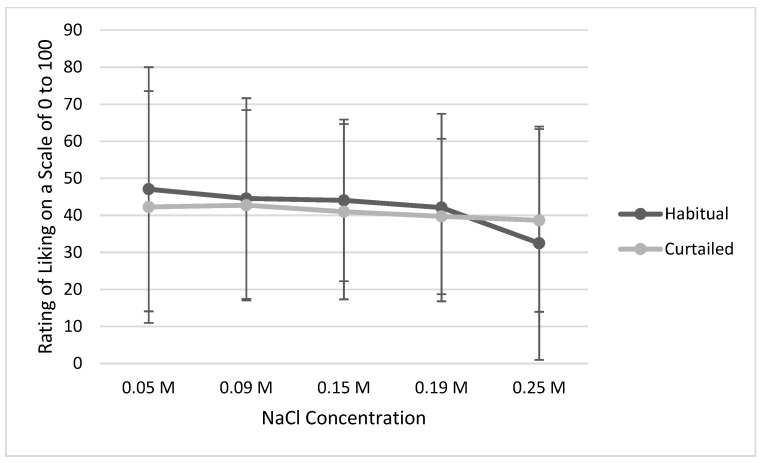
Mean liking ratings of salt solutions across all concentrations after the habitual vs. curtailed night. Note: The error bars represent standard deviations.

**Figure 3:**
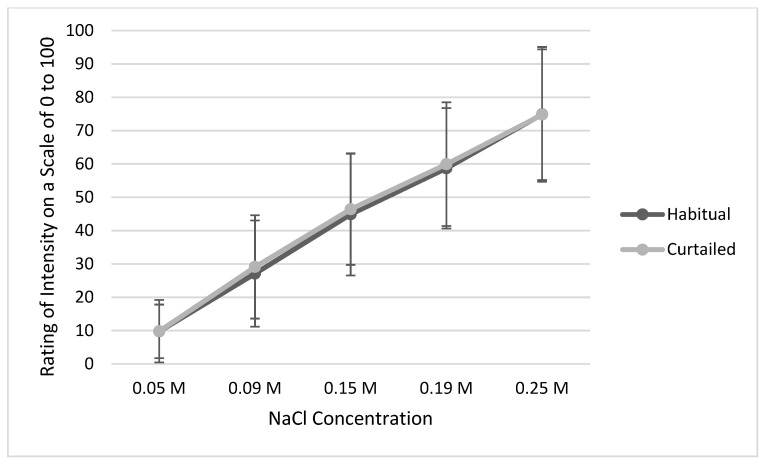
Mean intensity ratings of salt solutions across all concentrations after the habitual vs. curtailed night. Note: The error bars represent standard deviations.

Slopes of liking (*p* = 0.074, Figure 2) and intensity ratings (*p* = 0.844, Figure 3) of salt solutions were not different between the habitual and the curtailed nights of sleep. Further, neither preferred concentration of saltiness (habitual 0.12 ± 0.06 M vs. curtailed 0.13 ± 0.06 M, *p* = 0.092), sodium intake, nor energy-corrected sodium intake differed after the night of habitual sleep compared to the curtailed night (Table 4). In terms of dietary intake, energy consumption (kcal/d), carbohydrate (g/d), protein (g/d), fat (g/d), and Na (mg/d) intake were not different between sleep conditions.

### 3.5. Correlations between Hedonic Measures, Salt Taste Function, and Energy-Corrected Sodium Intake under the Habitual and the Curtailed Sleep Condition

In the habitual sleep condition, the liking slope was positively correlated with preferred concentration and energy-corrected Na intake. Whereas, after sleep curtailment, the association between the liking slope and energy-corrected Na intake no longer existed.

The liking slope was positively associated with higher preferred salt concentration and higher sodium intake after one night of habitual sleep but not after one night of curtailed sleep (Table 5).

Age, BMI, %BF, sleep quality and duration were not associated with hedonic measures of salt, intensity, or energy-correlated Na intake. Additionally, salt taste intensity and preferred salt concentration were not associated with energy-corrected Na intake.

Under the curtailed sleep condition, liking slope positively correlated with preferred salt concentration; however, sleep curtailment disrupted the relationship between liking and energy-corrected Na intake (Table 6). All other relationships did not change when compared to the habitual sleep condition, including the positive correlation between the liking slope and the preferred salt concentration.

In order to explore why sleep curtailment disrupted the relationship between liking and energy-corrected Na intake, standard deviations of liking ratings between the habitual and the curtailed night were compared to test whether the variance in liking ratings under the curtailed condition was increased. The results revealed that variances in liking ratings were larger only for the highest NaCl concentration solution (*p* = 0.032) after the curtailed night of sleep compared to the habitual night of sleep.

### 3.6. Exploration of Salt-Liking Phenotypes

**Figure 4:**
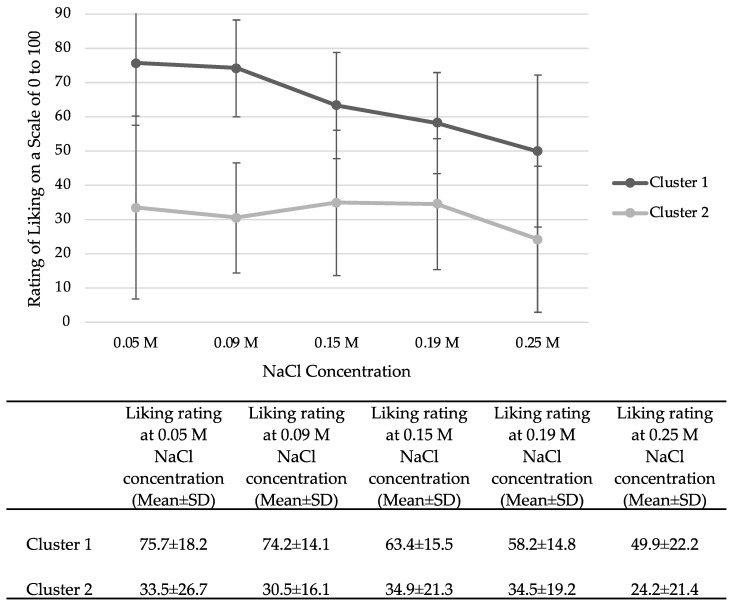
The graph shows the visual differences between the two clusters. Exact mean and SD values are shown in the table for reference. Salt-liking phenotypes based on hierarchical cluster analysis. Cluster 1: salt likers, *n* = 19; cluster 2: salt dislikers, *n* = 40. SD = standard deviation.

As there was no significant difference in salt-taste liking between the two sleep conditions, data from habitual sleep was used to explore salt-liking phenotypes. Two phenotypes were identified using hierarchical cluster analysis (Figure 4). Participants in cluster 1 expressed a higher level of liking compared to participants in cluster 2 for each concentration (*p* < 0.001 for all). The mean liking ratings of all concentrations in cluster 1 are above the midpoint of the VAS scale while mean liking ratings in cluster 2 are below the midpoint for all concentrations. Based on these results and following the conventions established in the sweet-liking phenotype literature, we propose that cluster 1 represents salt likers and cluster 2 represents salt dislikers. Due to the small sample size (*n* = 19) in the salt likers group, statistical comparisons between the two groups were not conducted.

## 4. Discussion

The purpose of the present study was to examine whether the adapted version of the forced-choice paired-comparison tracking procedure could be used as a reliable and valid tool for determining salt taste preference, and to evaluate the effects of sleep on salt taste hedonic measures and function. The results from the methodology validation component of the study demonstrated that the adapted forced-choice paired-comparison tracking procedure could serve as a reliable and valid test to determine salt-taste preference when compared to conventional methods. The randomized cross-over trial demonstrated there were no significant changes in salt-taste function or hedonic measures after one night of curtailed sleep compared to one night of habitual sleep. However, the slope of liking was associated with energy-corrected Na intake, but only under the habitual sleep condition. These results suggest that the lack of associations between salt-taste measures and dietary intake reported by the current literature [4,5] could be attributed, in part, to not accounting for sleep duration.

### 4.1. Validity of the Adapted Forced-Choice, Paired-Comparison Tracking Procedure in Determining Salt Taste Preference

The forced-choice, paired-comparison tracking procedure was originally developed to determine sweet taste preferences for adults [42], but the present study is the first to adapt and validate the same forced-choice paired-comparison tracking test procedures in assessing salt-taste preference. Three validation steps were undertaken. First, participants were able to distinguish the intensity of the salt solutions, hence demonstrating the appropriateness of the salt concentrations used in the test. Second, two trials to determine preferred concentrations were performed, and the concentrations selected for each trial were not significantly different from each other or from the geometric mean of the two trials. Additionally, more than 80% of participants picked either the same or neighboring concentrations for the first and the second trial, indicating the results were reproducible between the two trials. Third, the preferred salt concentration was positively associated with the liking slope, which suggests that the adapted forced-choice paired-comparison tracking test could be used in place of assessing liking slope, should the researcher wish to conduct only one hedonic measure. Given these outcomes, the present study supports the use of the adapted forced-choice paired-comparison tracking test in determining a preferred salt concentration.

One major limitation in sensory research is that there is no consensus on salt-taste test procedures, which makes compairing results from one study to another difficult [4]. For example, some studies measured salt liking on a 9-point hedonic scale, using 1 representing “dislike extremely” while 9 representing “like extremely” (e.g., [53]) while others measured liking using a general Labeled Magnitude Scale (e.g., [1]).

Liking is commonly evaluated with Likert scales or visual analog scales (VAS) [54]. For Likert scales, the options are usually limited to five to seven choices, which may not truly reflect the attitude or feeling of participants [55]. Additionally, it is arbitrary to translate the ordinal results of Likert scales into a continuous variable as is commonly done when analyzing Likert scale data [55]. In terms of VAS, even though such scales can provide continuous data, the common disadvantage of VAS is that participants may use the scale differently, for example, two participants may wish to indicate that the stimulus is liked “slightly,” but that same perception could be marked on very different locations on the scale [54]. Therefore, repeated measures testing is strongly recommended when using VAS so that each participant serves as their own control.

Methods for assessing preference are highly variable, making comparisons across studies challenging, and limitations to these methods exist. For example, some studies ask participants to add salt into broth until the preferred concentration is achieved (e.g., [14]) while others assess self-reported salt preference in foods (e.g., [15]) or frequency of consuming common salty foods (e.g., [56]). One advantage of using the concentrations delineated in the current study is that they have been shown to be distinguishable from each other and span a range of concentrations that encompass commonly encountered sensations when eating or drinking [50]. However, preferred concentration testing is limited in that it identifies only one concentration, which can be heavily influenced by the concentrations selected for testing as well as the differences between each concentration [57]. Therefore, if only relying on one test to assess preference, researchers should carefully consider which one best meets their needs in the context of the proposed research question.

When comparing liking slopes and preferred concentration approaches in determining salt-taste liking, the liking slopes use multiple data points to describe a function, which provides a more comprehensive understanding of taste responses over a range of concentrations. This more holistic evaluation could explain why energy-corrected Na intake was associated with liking slope but not preferred concentration. Results from the present study suggest that if a researcher is attempting to predict sodium intake from taste hedonics, the slope of liking responses is the better choice.

### 4.2. The Effects of Sleep on Salt Taste Function

Sleep curtailment did not affect salt-taste intensity assessments, which aligns with our hypothesis and agrees with what has been reported regarding the effects of sleep curtailment on sweet taste function [16,20,22,26,35,58,59,60,61,62]. Two previous studies examined the effects of sleep duration alteration on salt taste function; one implemented sleep deprivation [33] while the other investigated the effects of short sleep (<7 h) versus long sleep (>7 h) on salt taste function [34]. Both studies reported sleep duration had no effects on salt taste sensitivity; however, it is difficult to compare these results with ours because of the differences in taste measures assessed. The present study examined salt-taste sensitivity at a suprathreshold level using an intensity rating method while the other two studies focused on examining detection thresholds [33,34]. Detection thresholds, on the other hand, are assessed using salt concentrations that span a range from undetectable to detectable [35], with the goal to determine the lowest salt concentration that can be detected. Thus, detection threshold testing and intensity testing measure two different attributes of taste function [63]. In terms of the effects of sleep on sweet-taste function, previous studies demonstrated that sweet taste function, measured by both intensity rating [22,26] and detection threshold [34], did not differ after one night of curtailed sleep. Thus, current and previous findings suggest both salt- and sweet-taste function is relatively robust after one night of short sleep. Future work should focus on investigating the effects of chronic sleep curtailment on sweet- and salt-taste functions.

### 4.3. The Effects of Sleep on Hedonic Measures of Salt Taste

Contrary to our hypothesis, sleep curtailment for one night did not affect hedonic measures of salt taste, which contradicts our findings on the negative effects of curtailed sleep on sweet-taste hedonics [22,26]. This difference could be attributed to differences in the activation of the reward system of the brain by these two tastes [64]. Neural and behavioral reactivity to pleasurable experiences increase after sleep deprivation and curtailment [65]. For example, under conditions of shortened sleep, consuming sweets becomes more pleasurable after one night of short sleep; therefore, liking and consumption of sweets increase [66]. However, previous studies noted the anterior insula of the brain, which is the putative primary taste cortex [67], was activated more with NaCl than with sucrose solutions [64]. The anterior insula plays a role in negative valence-specific responses in taste [68], responding more to taste that is unpleasant. Salt solutions are often rated as less pleasant compared to sucrose solutions [64]. Additionally, under sleep deprivation or curtailment, the ability for the anterior insula to discriminate reward versus punishment decreased [69,70,71]. Given the anterior insula is activated more for salty taste and is less sensitive under short sleep conditions, acute sleep curtailment may not increase the liking of salt. In summary, differences in the effects of sleep on salty vs. sweet hedonics could be because of differences in the valence and neural activity produced by these tastes.

### 4.4. Associations between Salt Taste Measures and Dietary Intake

Curtailed sleep eliminated the expected relationship between salt taste liking and energy-corrected Na intake, likely due to curtailed sleep contributing to greater variance in salt liking of the highest concentration. We noted that participants reported a significantly higher variability in liking ratings only for the highest NaCl concentration solution (1.46 g NaCl/100 mL), which is within the range of high salt foods. For example, NaCl concentration of pickle juice is 3.18 g NaCl per 100 mL, which exceeds the highest concentration tested [72]. Sleep curtailment has been shown to negatively affect the prefrontal cortex of the brain, which decreases decision-making and self-monitoring abilities [73,74]. This may explain why liking ratings varied more under the curtailed sleep condition; however, why only ratings of the highest NaCl concentration solution were affected is unknown. Further exploration regarding the relationship between taste measures and diet intake under curtailed sleep conditions is needed.

The association between salt-taste liking slope and energy-corrected Na intake was only present under the habitual sleep condition, which provides one possible explanation for the lack of associations between taste measures and dietary intake reported in the literature [4,5]. Almost all studies investigating the relationships between taste measures and dietary intake fail to account for sleep curtailment or deprivation [4,5]. In the present study, we observed that the association between salt-taste liking slope and energy-corrected Na intake disappeared after one night of curtailed sleep, which suggests that sleep curtailment disrupted the previously observed association between liking and intake. Given that one in three adults worldwide do not routinely achieve adequate sleep [75,76,77,78] failing to account for short sleep duration could obscure taste test-dietary intake relationships. Therefore, the findings from the present study suggest that sleep duration should be considered when investigating relationships between taste and diet.

### 4.5. Identification of Salt-Liking Phenotypes

Salt-liking phenotypes, including salt likers and salt dislikers, were detected. Individuals who rated liking of all sodium concentrations above the mid-point of the liking scale were identified as “salt likers” while “salt dislikers” rated likings of all solutions below the mid-point. Both salt-liking phenotypes demonstrated a decline in liking as concentrations of sodium increased.

The salt-liking phenotypes identified in this study differ from the established sweet-liking phenotypes reported in the literature, e.g., [38,39,40,79]. Sweet-liking phenotypes reliably identify sweet “likers” who show increased liking as concentrations of sweeteners increase; sweet “dislikers” who report decreased liking as concentrations of sweeteners increase; and “inverted U-shape” responders, whose liking increases and then falls as the concentrations of sweeteners go above the most liked concentrations. Salt “liker” and “disliker” liking patterns followed the same curve but were at different places on the scale. The reasons why salt-liking and sweet-liking phenotypes are different warrant further exploration, particularly using real foods, as aqueous salt solutions are not routinely consumed; whereas, some sweet taste exposures are fairly limited in terms of sensory input, e.g., sugar sweetened beverages.

### 4.6. Strengths and Limitations

This work has several strengths. First, the study included objective sleep data to validate sleep duration and verify compliance to sleep protocols. Second, both salt-taste function and hedonic measures were examined, which provide a more comprehensive evaluation of salt taste. Third, sleep curtailment was individualized based on habitual sleep duration of participants, which ensured the consistency of sleep curtailment between participants. Fourth, the study incorporated a one-week wash-out period between the two taste test sessions for study 2 to avoid carry-over effects. Finally, this project employed a randomized cross-over design, the gold standard for experimental rigor.

Several limitations are also noted. Results from this work may have limited generalizability, as mostly young adults were tested. Future studies should consider testing the forced-choice paired-comparison tracking procedure in other populations, such as teenagers and older adults. Additionally, the study included only testing short-term sleep curtailment, and only one 24-h dietary recall was obtained after the habitual and curtailed nights of sleep; hence, this may not represent habitual intake. While menstrual cycle status was not accounted for, studies of young, healthy women report limited effects on total sleep time [80] or sleep quality throughout the cycle [81]. Taste and intake changes of women measured over the course of three months reported no difference in sodium intake or salt-taste sensitivity between the luteal and follicular phases [82]. If there were variations in sleep or taste based on menstrual cycle status, the randomization used should have helped to control for these effects. Some taste measures, such as sensitivity, appear to be robust to acute sleep curtailment; however, future studies should consider investigating the effects of chronic sleep curtailment on salt-taste measures. Future research should consider using multiple dietary assessment methods and/or consider using indirect methods of measuring intake, such as urinary sodium. In addition, studies should focus on the effects of sleep curtailment on salt-taste measures among salt likers and dislikers in the future.

## 5. Conclusions

The present study demonstrated that the forced-choice paired-comparison tracking procedure is a reliable and valid tool for determining salt taste preference. Results also indicated that acute sleep curtailment did not affect salt taste function or hedonic measures; however, the expected relationship of salt-taste liking and energy-corrected Na intake was disrupted after sleep curtailment. These findings suggest that sleep duration should be considered in taste studies examining relationships between taste and dietary intake. Salt-taste measures appear to be robust after acute sleep curtailment; therefore, future studies should consider examining the effects of chronic sleep curtailment on salt taste. Further, researchers should consider adopting the NaCl concentrations used in the present study for salt-taste testing and use the forced-choice paired-comparison tracking tool as a standard procedure to measure salt taste preference to facilitate direct comparisons between future taste studies. Finally, two salt-liking phenotypes were detected in the present study. Replication of salt-liking phenotypes and, if present, exploration of their importance to human health is warranted.

## Figures and Tables

**Table 1:** Anthropometric and demographic summary.

Sex	N	%
Male	18	30.5
Female	41	69.5
Race		
Asian	23	39.0
Black	5	8.5
White	27	45.8
Other	3	5.1
Ethnicity		
Hispanic	2	3.4
Non-Hispanic	51	86.4
Anthropometrics	Mean ± SD	Range
Body mass index (kg/m^2^)	23.3 ± 4.4	15.5–36.6
Body fat (%)	24.7 ± 10.8	3.0–49.5
Age (y)	26.2 ± 6.0	19–41
Sleep measures	Mean ± SD	Range
Pittsburg Sleep Quality Index (score)	4.0 ± 1.7	0–8
Sleep duration (h, self-reported)	8.1 ± 0.6	7.1–9.1

Race missing *n* = 1, ethnicity missing *n* = 6, body fat % missing *n* = 1.

**Table 2:** Comparison of preferred salt concentration between participants who experienced the habitual sleep night first versus second.

	Geometric Mean of the Preferred Salt Concentration for Those Who Experienced the Habitual Sleep Night First (*n* = 27) (Mean ± SD)	Geometric Mean of the Preferred Salt Concentration for Those Who Experienced the Habitual Sleep Night Second (*n* = 32) (Mean ± SD)	Geometric Mean of the Preferred Salt Concentration after the Habitual Sleep Night for the Overall Sample (*n* = 59) (Mean ± SD)	*p* Value for the Comparison of the Habitual Sleep Night Experienced as the First Night vs. the Second Night
Habitual sleep night	0.71 ± 0.38 mg/dL	0.70 ± 0.36 mg/dL	0.70 ± 0.37 mg/dL	0.920

**Table 3:** Summary of objective and subjective sleep measures (*n* = 59).

		Habitual	Curtailed	% Reduction	*p*-Value
Objective sleep measures (h)	Time in bed	8.6 ± 0.8	5.5 ± 0.7	36.0%	< 0.001
	Total sleep time	7.2 ± 0.6	4.6 ± 0.7	36.1%	< 0.001
	SWS sleep	1.5 ± 0.4	1.3 ± 0.5	13.3%	0.002
	REM sleep	1.8 ± 0.6	1.1 ± 0.4	38.9%	<0.001
Subjective sleep measures (0–100 scale)	Sleep quality satisfaction	64.1± 17.5	41.4 ± 20.4	35.4%	<0.001
	Sleep duration satisfaction	67.9 ± 17.6	27.3 ± 14.1	59.8%	<0.001

SWS = slow wave sleep; REM = rapid eye movement sleep.

**Table 4:** Energy, macronutrient, and sodium intake following habitual and curtailed sleep conditions (*n* = 59).

	Habitual	Curtailed	*p*-Value
Na (mg/d)	3251.9 ± 1866.6	3156.9 ± 1400.0	0.621
Energy-corrected Na (mg/Kcal)	1.7 ± 0.5	1.7 ± 0.6	0.986
Energy (Kcal/d)	1984.3 ± 1101.6	1948.6 ± 818.0	0.757
Carbohydrate (g/d)	249.0 ± 156.7	235.6 ± 120.3	0.454
Protein (g/d)	73.5 ± 54.0	70.8 ± 37.7	0.657
Fat (g/d)	76.7 ± 47.5	77.6 ± 36.9	0.864

**Table 5:** Zero-order correlations under the habitual sleep condition.

Measures	1	2	3	4	5	6	7	8	9
(1) Age (years)	-	0.213	0.295 ^†^	−0.087	−0.138	−0.129	0.161	−0.078	−0.239
(2) BMI (Kg/m^2^)		-	0.798 **	0.012	−0.029	−0.004	0.098	−0.001	0.021
(3) BF%			-	−0.046	0.046	−0.110	0.121	−0.030	−0.119
(4) PSQI				-	0.020	0.072	−0.174	0.177	−0.154
(5) Total sleep time (h)					-	−0.104	−0.248	0.075	0.024
(6) Liking slope						−	0.009	0.593 **	0.338 **
(7) Intensity slope							-	−0.199	0.005
(8) Preferred salt concentration (M)								-	0.165
(9) Energy-corrected Na intake (mg/Kcal)									-

** *p* < 0.01, ^†^ no longer significant after the FDR adjustment.

**Table 6:** Zero-order correlations under the curtailed sleep condition.

Measures	1	2	3	4	5	6	7	8	9
(1) Age (years)	-	0.213	0.295 ^†^	−0.087	−0.084	−0.315 ^†^	0.158	−0.021	0.037
(2) BMI (Kg/m^2^)		-	0.798 **	0.012	−0.214	0.074	0.294 ^†^	−0.014	0.217
(3) BF%			-	−0.046	−0.195	−0.119	0.259 ^†^	−0.170	0.077
(4) PSQI				-	−0.061	0.136	0.162	0.060	0.203
(5) Total sleep time (h)					-	−0.171	−0.259 ^†^	−0.032	−0.255
(6) Liking slope						−	−0.029	0.671 **	0.132
(7) Intensity slope							-	−0.099	0.299 ^†^
(8) Preferred salt concentration (M)								-	−0.016
(9) Energy-corrected Na intake (mg/Kcal)									-

** *p* < 0.01, ^†^ no longer significant after the FDR adjustment.

## Data Availability

Data are available from the corresponding author once all analyses are complete.
